# Promoting Fundamental Movement Skills and Physical Literacy Among 8–12-Year-Old Children: Feasibility Insights from an 8-Week Pilot Program in Southwestern Ontario

**DOI:** 10.3390/children12070838

**Published:** 2025-06-25

**Authors:** Danielle Salters, Emily Chauvin, Sarah J. Woodruff, Sara M. Scharoun Benson

**Affiliations:** Department of Kinesiology, University of Windsor, Windsor, ON N9B 3P4, Canada; chauvin8@uwindsor.ca (E.C.); woodruff@uwindsor.ca (S.J.W.)

**Keywords:** game-based, intervention, physical activity, fundamental movement skills, physical literacy, participation

## Abstract

**Background/Objectives**: Global levels of physical activity are in decline, accompanied by low levels of competence in fundamental movement skills (FMSs) required to meaningfully participate in lifelong physical activity. This study aimed to explore the effects of an 8-week pilot program on the development of FMSs for children in a lower socioeconomic area through a game-based physical activity approach. **Methods**: Children (*N* = 30) were recruited from a lower socioeconomic area to participate in a community-offered PA program. This 8-week pilot program focused on game-based intervention, with weekly 1.5 h sessions to promote active play and development of FMSs. Each session was structured to ensure at least one FMS based on the Test of Gross Motor Development—Third Edition (TGMD-3) was emphasized to promote practice in these skills. The TGMD-3 was employed as a pre- and post-test measure of motor competence. **Results**: Children who completed both the pre- and post-test assessments (*N* = 11) demonstrated improvements in both locomotor (*p* = 0.166) and ball skill (*p* = 0.184) scores, though these were not significant. Additional analyses at baseline with all participants (*N* = 22; 8 not present at baseline testing) were insignificant, but descriptive statistics demonstrated that boys scored higher in ball skill scores, while girls scored higher in locomotor skill scores. Older children at baseline were found to score significantly higher than younger children in ball skill scores. **Conclusion**: The results highlighted that the game-based intervention did demonstrate some improvements for FMSs, highlighting a need for further development of the program and the participation of more children for more strongly powered analysis and to account for program withdrawal or dropout.

## 1. Introduction

Fundamental movement skills (FMSs) are organized movement patterns categorized as locomotor, object control and manipulation, and balance and stability skills [1]. FMSs are an integral component to the development of overall physical literacy [2], which encompasses the different aspects involved with meaningful participation in physical activity (PA). This includes the motivation, confidence, and competence to participate in a wide range of activities [2].

Globally, FMS competence is in decline. Children are failing to achieve age-related benchmarks in proficiency beyond “average” levels of competence [3], despite the developmental potential to achieve mastery by the age of 6 to 8 years [4,5]. Likewise, fewer than 20% of adolescents, globally, are meeting recommended PA guidelines [6]. FMS competence and overall physical literacy have been found to be positively associated with increased PA, not only during childhood but throughout adolescence and into adulthood [7,8,9]. While FMS competence and physical literacy develop at different rates based on age, instruction, and context [5,7], this underscores a need for interventions that promote FMSs and physical literacy required for PA.

There have been numerous approaches and interventions designed to promote FMS development, including video games/virtual reality [10], specific focus on instructors/teachers and parental inclusion [11,12], and sport/activity-specific intervention designs [13,14]. Evidence also suggests that game-based approaches (GBAs) are effective for the development of FMS competence and promoting participation in PA [15,16]. These interventions situate learning within game environments, with instructors as facilitators for individual learning [16]. Here, GBAs refer to structured/semi-structured, play-oriented PA, designed to target FMSs in a fun, engaging environment.

The effects of GBAs on FMS development are not widely researched [17]. A recent systematic review [16] on GBAs found only 3 of 17 included studies directly measured FMS development as an intervention outcome, while the other studies assessed other outcomes, such as motivation, decision-making, PA, and game play [16]. This demonstrates a need to further investigate how GBAs can be adapted for broader, community-based delivery, beyond sport and physical education. GBAs have been effective in sport-specific contexts to improve both athletic ability and overall FMSs [15]. However, the effects have been shown to only work in the short term, with evidence highlighting that intervention effects on FMSs have not been sustained within a 4-to-8-week retention period; more sustained effects have been found with more frequent intervention sessions, including an increased amount of time for practice and participation [15].

The effects of GBAs have traditionally been studied in physical education environments [15,16,17,18,19]. In fact, GBAs were initially introduced under the “Teaching Games for Understanding” model [20] as a method to improve skills and performance of students in game situations. GBAs have been found to be beneficial in several ways, the most notable of which are improved FMSs (particularly object control skills) and higher levels of enjoyment and motivation to participate [16,17], which are essential components of physical literacy [2]. Though often school-based, GBAs reflect the characteristics of deliberate play in interventions outside of the school system [21], generally requiring simplified rules and a majority of time dedicated to play and practice [19]. This approach has demonstrated mixed results, with interventions focusing solely on deliberate play (e.g., game play) demonstrating less effective contributions to skill development compared to deliberate practice or combined interventions [21].

This highlights a need to further explore the impact of GBAs on FMSs and physical literacy. GBAs provide a focus on technical competency (i.e., skill competence) rather than tactical strategies for game play, which should be a primary focus on early development of FMSs [16,22]. A foundation of FMSs is critical to long-term participation in PA [7,8], underscoring the necessity of research on non-sport-specific GBA designs that take place outside of the school context.

Intervention designs for FMS development employ a number of methodological and theoretical approaches [3,9,21,23]. Evidence-based interventions designed to promote FMS development demonstrate that both short-term (4 to 8 weeks) and long-term (longer than 8 months) intervention designs have significant effects on skill development [23,24]. Despite these differences, it is widely accepted that early intervention is key, and that promoting the development of FMSs through structured activities is critical to promoting lifelong participation in PA.

### Purpose/Aim

Taken together, research supports the role of motor competence in promoting long-term PA. However, much of the FMS research has taken place in school settings [9,21,24]. Few interventions have been implemented in underserved, community-based environments. Furthermore, GBAs have not been extensively used in community-based research, demonstrating a need to explore the use and efficacy of this approach. This study aimed to address this gap by exploring the feasibility and pilot effects of an 8-week intervention designed to promote FMS development and physical literacy for children in a lower socioeconomic area and determine if this led to improvements in motor competence. The current study applies EST to provide a multi-level approach to developing FMSs and promoting physical literacy. By addressing individual, social, institutional, and cultural influences on participation, this pilot program aimed to promote the development of FMSs through meaningful engagement with PA using a game-based approach. The following research questions were used to guide this research: (1) To what extent does participation in an 8-week game-based intervention influence children’s FMSs in a community PA program? (2) What were the effects of program duration and design on skill acquisition? As an exploratory pilot study, no hypotheses were proposed.

## 2. Materials and Methods

### 2.1. Theoretical Approach

The present research is grounded in Bronfenbrenner’s Ecological Systems Theory (EST) [25,26]. In this research, EST was used to inform both the structure of the program (i.e., providing a free program to an underserved community with little funding available) and the evaluation of the program. Through this framework, EST emphasizes that FMSs and physical literacy are not solely dependent on the individual but are shaped by social, institutional, and cultural factors [25,26].

With over 80% of adolescents, globally, failing to meet the recommended PA [6], children from low socioeconomic communities may be disproportionately affected. Those with a lower socioeconomic status typically engage in less active play compared to individuals from higher socioeconomic areas due to limitations such as access to play space and organized programming [27,28]. While lower socioeconomic status may not negatively impact FMS competence, the relationship between PA and FMS competence is widely established [7,8,9], emphasizing the importance of access to PA programming. The pilot program employed in this research recruited participants from a lower socioeconomic area with one of the highest poverty rates in Canada according to the 2016 Census [29]. Additionally, this area has seen rapid population growth (an 11.3% increase from 2016 to 2021) [30], with over 43.5% of the community identifying as a visible minority. This relates to the macrosystem of EST, providing free access for children in a lower socioeconomic area in order to remove the financial barriers associated with participation in PA.

### 2.2. Participants

Participants were 8- to 12-year-old children recruited from a city in Southwestern Ontario, in an area classified as having a lower socioeconomic status [29,30]. Potential participants were recruited from local schools and community postings. Given the pilot nature of the program, minimal funding, and capacity considerations (i.e., average ratio of 1:2 instructors per children), enrolment was intentionally capped at 30 children. Research supports an enrolment cap of 30, with recommendations for pilot studies including sample sizes of 24–50 participants for feasibility studies such as the current research, focusing on refining intervention planning and implementation [31]. No statistical power analyses were conducted for the present research due to the exploratory, feasibility-focused nature of this pilot program; sample size for this program were pragmatically determined based on program constraints. As this was an open PA program offered to the community, enrolment was offered on a first-come, first-serve basis, with a waiting list offered for other children. All children who met the program criteria (i.e., between the ages of 8 and 12 years) were eligible to participate in the intervention. Informed consent was obtained from parents/guardians of the children through the permission forms signed upon enrolment. Verbal assent was further collected by the children on testing days. This included a mix of girls (*N* = 7; M_age_ = 9.9 ± 1.55) and boys (*N* = 19; M_age_ = 9.8 ± 1.14).

Twenty-six children attended sessions and four did not. Of those that attended sessions, 22 completed baseline evaluations, and 14 completed post-test evaluations. Of those who completed the baseline evaluation, 11 completed the post-test and attended at least 6 of the 8 sessions (3 attended all sessions, 7 missed one session, and 1 missed two sessions). Three children enrolled after baseline testing and only completed the post-test. Eleven children completed the baseline evaluation but dropped out and did not complete the post-test evaluation, resulting in a 42% dropout rate. Children who dropped out after the first session either did so following the first two weeks (*N* = 5) or did not come to the final two weeks (*N* = 6); reasons for dropout were not collected as this was an open program focused on providing access to PA to the community. In total, 11 children completed both pre- and post-test evaluations, including 3 girls (M_age_ = 9.45 years) and 8 boys (M_age_ = 10.14 years).

### 2.3. Measurement Tools

The Test of Gross Motor Development—3rd Edition (TGMD-3) [32] was used to assess FMS competence at baseline and post-intervention periods. The TGMD is one of the most frequently used assessment batteries for FMS [33], with the most recent edition demonstrating high levels of reliability (above 0.89) [34]. The TGMD-3 consists of 13 skills, including 6 locomotor skills and 7 object control skills. Stations were set up for each skill, and assessors were assigned one locomotor skill and one ball skill test that they assessed at both baseline and post-test evaluations to ensure consistency. Children cycled through the locomotor and ball skill stations in a circuit format. Assessors demonstrated the skills, followed by a practice trial for each child before two test trials were completed and scored.

The TGMD-3 is intended for use among children aged 3 years 0 months to 10 years 11 months [32,33]. The current population did include some 11-year-old children (*N* = 4, though none exceeded the age of 12 years); however, the average age of older participants was 10.94 years, falling within the acceptable age range for the TGMD-3. Recent data have indicated that the TGMD could be used with adolescent populations [35], and use of the TGMD with children in the 11–12 age range has been employed in additional peer-reviewed research [36]. As such, the decision was made to use the TGMD-3 for all participants.

### 2.4. Intervention

Ethics clearance was obtained from the institutional Research Ethics Board (REB# 23-107). The pilot program was designed as a community-based PA program to provide children in a lower socioeconomic area with semi-structured PA to promote physical literacy and FMSs. Given minimal funding, the program was designed to be small-scale (i.e., 30 participants), with sustainability and scalability in mind. This study employed a single-group, quasi-experimental design to assess the feasibility and pre–post effects of this pilot program.

Instructors (*N* = 14) were recruited from undergraduate classes in the Department of Kinesiology to facilitate activities and assess TGMD-3 components. Instructors included undergraduate students, with two group leaders from 3rd and 4th year classes, a coordinator from a 4th year class, and the rest from the 2nd or 1st year of undergraduate studies.

The principal investigator (author 1) delivered two 1.5 h training modules that included background, discussion, and practical application of concepts. The first module emphasized understanding FMSs and physical literacy, including the main tenets, the importance for growth and development and long-term health, and potential instructional strategies to promote skill development. The second module focused on TGMD-3 training [37]. A general overview of the TGMD-3 was given, and instructors were guided through training videos to practise coding the various skills and were given practical time to practise both in and out of the training session. Upon completion, instructors were assigned one locomotor and one ball skill test to ensure all skills at baseline and post-test evaluations were consistently assessed by the same individual(s). This resulted in two assessors per locomotor and ball skill test to enhance consistency in skill scoring.

The PA program was offered as an accessible opportunity for children in a lower socioeconomic area in Southwestern Ontario; as this was the first offering of the program, it was deemed inappropriate to withhold access to the program from some children. Additionally, the decision not to include a control group was influenced by logistical constraints, including access to resources, scheduling, and instructor availability. Enrolment was capped at 30 children to maintain an average ratio of instructors to children of 1:2 (based on those who attended the sessions) and promote engagement and feedback. Prior to data collection and the onset of the program, consent was collected from parents/guardians of those who registered for the program. Child assent was verbally collected during data collection for the TGMD-3. The first and last sessions of the program included dedicated time for administration of the TGMD-3. TGMD-3 testing sessions were structured in a circuit-style activity, where assessors remained at specific stations to score their assigned skill, and children rotated to ensure they completed all tasks.

Children were divided into two pre-determined groups (older: 10–12 years old, and younger: 8–9 years old) based on child age at enrolment, with 7 instructors assigned to the younger age group and 6 assigned to the older age group. Table 1 highlights the basic demographic (e.g., age/gender) distributions for each group. The PA program was offered once per week for 90 min over an 8-week period. The program took place in an indoor, open-recreation space. The program coordinator (author 2) planned the program to promote physical literacy, through a focus on FMS development. Structured skill development activities were based on “PLAYBuilder”, a list of activities offered through Sport for Life Canada, and focused on developing specific skills through a GBA. A breakdown of the weekly activities by age group can be found in the Supplementary Files. Each session was structured to promote the development of at least one FMS assessed through the TGMD-3 (e.g., dribbling, kicking, hopping, etc.), while allowing opportunities to consolidate learning and development from previous sessions. Sessions followed a GBA to skill development and included a variety of games (4–6 games per session), which consisted of a quick warm up (10–15 min), followed by skill-focused activities, and concluding with child-led games. This allowed for meaningful organization of activities and differentiated strategies and activities to promote skill development throughout the session. Instructors employed a variety of instructional strategies, including, but not limited to, instructor and peer-modelling, team/cooperative game play, and independent practice. The two groups engaged in different activities based on developmentally appropriate behaviours (e.g., increased distance or difficulty for the older child group) but still had a focus on the same skill.

### 2.5. Data Analysis

Data were analyzed using JASP statistical software (version 0.18.1). TGMD-3 raw scores, percentile ranks, and age equivalents (based on TGMD-3 conversion tables) were used to calculate scaled scores and gross motor quotient (GMQ). Scaled scores are used to provide comparisons between locomotor and ball skill subtests. Percentile ranks are used to indicate the distribution around a particular score. Age equivalent data provide an indication of how subtest scores compared to typical age of children. Finally, the GMQ provides information about the relative motor competence of the individual from the tabulated scaled scores [32,33]. All scores were calculated prior to data analysis. Reliability measures were run for the TGMD-3 at baseline conditions. Reliability assessments included both baseline and post-test raw skill scores, subtest raw scores, subtest scaled scores, and GMQ. The TGMD-3 demonstrated moderate to high levels of internal consistency, as defined by a Cronbach’s alpha of 0.887 (CI = 0.824–0.934). Correlations were conducted to assess the relationship between TGMD-3 subtests (e.g., locomotor and ball skills, raw skills, scaled scores, and GMQ) to determine associations between locomotor and ball skill scores, as well as overall GMQ. Given the interconnected nature of motor skills and the contribution of both locomotor and ball skills scores to overall GMQ, correlations were analyzed to explore the associations among raw and composite skill scores in the TGMD-3 subtests. Pearson correlations were used to assess the strength and direction of associations.

Due to the small sample size of children who completed both baseline and post-test evaluations (*N* = 11), data were analyzed using a paired-samples *t*-test. Two paired-samples *t*-tests were conducted. First, baseline and post-test evaluations were compared based on subtest and GMQ scores. Baseline locomotor and ball skills were compared using raw skills and scaled skill scores, and post-test locomotor and ball skills were similarly compared; baseline and post-test scores were compared for locomotor and ball skills using raw skills and scaled skill scores, and baseline and post-test GMQ were compared. The independent variable was the within-subject factor of time (baseline to post-test), with dependent variables of the subtest and composite scores obtained through the TGMD-3 at both time points. To control for potential Type I errors due to multiple comparisons (i.e., 6 comparisons were made in this analysis), a Bonferroni correction was applied. The adjusted alpha level was calculated as *p* < 0.01.

A second paired-samples *t*-test was conducted to explore the baseline and post-test performance on the individual raw skill scores. This pairwise comparison was only conducted with those who completed both baseline and post-test evaluations (*N* = 11). As the sample was so small for the pilot data, only baseline and post-test scores were considered for the pairwise comparisons, and no grouping variables (e.g., age, gender, etc.) were employed. The independent variable was the within-subject factor of time (baseline to post-test), while the dependent variables were the raw scores for each TGMD-3 skill.

Due to the small sample size of individuals who completed the entire intervention, no covariates were entered in the paired-samples *t*-test, and a focus of feasibility and initial outcomes were assessed. The baseline and post-test analyses were conducted using complete case data (e.g., only children who completed both the baseline and post-test assessments). No imputation techniques were employed due to the nature of the TGMD-3 and the exploratory nature of the study.

Secondary analyses emerged because of the unexpected dropout. Analyses were performed to assess the between-group characteristics at baseline of those who participated in the entirety of the intervention (*N* = 11) and those who dropped out (*N* = 15) to determine if there were any significant differences in motor competence prior to participation in the program. All data sets (i.e., TGMD-3 assessments) were complete, and all children’s data at baseline were included in between-group comparisons. Three multivariate analysis of variance (MANOVA) tests were run to assess differences based on child gender, the age groups, and whether they dropped out or completed the program. The independent variables were gender (boy and girl), age (older and younger), and dropout status (completed vs. completed). The dependent variables were the locomotor and ball skill raw subtest scores and GMQ derived from the TGMD-3 at baseline testing. Due to the distribution of participants (i.e., less than 3 observations per grouping), no interactions (e.g., pre–post x age x gender) were measured. Dependent variables that were assessed were the baseline scores for the locomotive raw subtest, the ball skill raw subtest, and the GMQ within the TGMD-3.

## 3. Results

### 3.1. Correlations

Correlations among TGMD-3 subtests can be found in Table 2. Several subtests demonstrated relatively strong, positive correlations, including baseline locomotor raw and scaled scores (*r* = 0.656, *p* < 0.001), baseline locomotor raw and post-test ball skill raw scores (*r* = 0.829, *p* < 0.01), baseline locomotor scaled scores and baseline GMQ (*r* = 0.664, *p* < 0.001), and post-test ball skill scaled scores and post-test GMQ (*r* = 0.888, *p* < 0.001). Other significant scores were found, with the ones outlined previously demonstrating the highest level of significance.

### 3.2. Pre–Post-Intervention Scores

Baseline and pre-test average scores can be found in Table 3. Data are based on mean (M) ± standard deviation (SD), unless explicitly stated. Change scores demonstrate the greatest improvements in locomotor raw skill scores (M = 3.27; SD = −3.15), ball skill raw scores (M = 2.91; SD = −1.64), and total raw skill scores (M = 6.18; SD = −4.79).

A paired-samples *t*-test assessed mean differences between baseline and post-intervention competence in the TGMD-3 subtests and overall GMQ. There were no outliers in the data; however, assumptions of normality were violated for all but three pairs (pre-test locomotor raw skill scores–pre-test ball skill raw scores; pre-test locomotor raw skill scores–post-test locomotor raw skill scores; post-test locomotor raw skill scores–post-test ball skill raw scores), as assessed by the Shapiro–Wilk test (*p* < 0.05 for all pairs); as such, the Wilcoxon signed-rank test was interpreted as a nonparametric measure. All paired comparisons can be found in Table 4.

No significant differences were found for any of the pairwise comparisons (*p* > 0.01), demonstrating no significant mean increases in subtest scores as an effect of the intervention. Most notably, comparisons with the highest change scores did not demonstrate significant differences: pre-test–post-test locomotor raw skill scores, *t*(10) = −1.494, *p* = 0.166, and pre-test–post-test ball skill raw scores, *z* = −1.631, *p* = 0.113.

A paired-samples *t*-test was additionally run to assess mean differences between baseline and post-intervention competence in the raw skill scores evaluated through the TGMD-3. There were no outliers in the data, and assumptions of normality were not violated as assessed by the Shapiro–Wilk test (*p* = 0.097–0.654) for all but three skills. Normality assumptions were violated (e.g., *p* < 0.05) for the hop, slide, and catch skills; these skills were assessed and interpreted using the Wilcoxon signed-rank test as a nonparametric measure due to these violations. Four skills were approaching significance: run, *t*(10) = 3.146, *p* = 0.010; slide, *z* = −2.380, *p* = 0.018; skip, *t*(10) = −2.104, *p* = 0.062; and overhand throw, *t*(10) = −3.985, *p* = 0.003. All pairwise comparisons for baseline and post-test scores for raw skills can be found in Table 5.

### 3.3. Between-Group Comparisons at Baseline

#### 3.3.1. Gender

A one-way MANOVA examined the differences in gender (boy and girl participants) of three dependent variables: locomotor raw skills, ball skill raw skill scores, and GMQ. Prior to analysis, assumptions of normality, linearity, and homogeneity of covariance were tested. Box’s M test was found to be non-significant (*p* = 0.424), indicating that the assumption of homogeneity of covariance was met, as well as a Shapiro–Wilk test of normality (*p* = 0.628). The MANOVA did not reveal a statistically significant effect of gender on the combined dependent variables, F(1, 3) = 0.864, *p* = 0.439. This suggests that gender did not significantly influence object control, locomotor skills, or GMQ.

Although the MANOVA was not significant, exploratory one-way ANOVAs were conducted for each dependent variable separately. The results indicated that there were no significant differences based on gender across the three different measurement conditions: locomotor raw skills, F(1, 20) = 0.059, *p* = 0.810, η^2^ = 0.003; ball skill raw skills, F(1, 20) = 0.423, *p* = 0.523, η^2^ = 0.021; GMQ, F(1, 20) = 1.678, *p* = 0.210, η^2^ = 0.077. Descriptive analyses were conducted to further explore group differences in locomotor skills, ball skills, and GMQ. Means and standard deviations for each dependent variable across gender groups are presented in Table 6. Girls scored slightly higher than boys in locomotor skills (M_diff_ = 0.77 points) and overall GMQ (M_diff_ = 2.27 points), while boys scored higher in object control skills (M_diff_ = 2.06 points). Effect sizes (η^2^) were computed to assess the magnitude of group differences. Small effect sizes were observed for locomotor skills (η^2^ = 0.003), ball skills (η^2^ = 0.021), and GMQ (η^2^ = 0.077), suggesting that gender differences among the current sample did not demonstrate practical importance.

#### 3.3.2. Age

A one-way MANOVA was run with older and younger age groups on locomotor skills, ball skills, and GMQ. Tests of normality, linearity, and homogeneity of covariance were conducted; Box’s M test (*p* = 0.654) and the Shapiro–Wilk test (*p* = 0.628) demonstrated assumptions of normality. MANOVA results indicated that the age differences between participants did demonstrate statistically significant effects on scores in the TGMD-3 (F(1, 3) = 18.0, *p* < 0.05). One-way ANOVAs were conducted for each dependent variable. The results further indicated that there were no significant differences based on age across locomotor raw skills (F(1, 20) < 0.001, *p* = 0.986, η^2^ < 0.001) and GMQ (F(1, 20) = 1.399, *p* = 0.251, η^2^ = 0.065). One-way ANOVA did demonstrate significant effects of age on ball skill raw skills (F(1, 20) = 11.515, *p* < 0.01, η^2^ = 0.365).

Descriptive analyses were conducted to further explore group differences in locomotor skills, ball skills, and GMQ. Means and standard deviations for each dependent variable across age groups are presented in Table 6. Older participants scored significantly higher in object control skills (M_diff_ = 7.99 points) and higher, although not significantly so, in overall GMQ (M_diff_ = 1.87 points), while younger children scored slightly higher in locomotor skills (M_diff_ = 0.25 points). Small effect sizes were observed for locomotor skills (η^2^ < 0.001) and GMQ (η^2^ = 0.065), suggesting that differences in these skills based on age did not demonstrate practical importance. A moderate effect size was found for ball skills (η^2^ = 0.365), demonstrating that age differences in ball skills may be important to explore in further investigations.

#### 3.3.3. Program Completion

The differences in baseline locomotor, ball skill, and GMQ scores were assessed for those who completed both the baseline and post-test conditions and those who completed only the baseline test condition through a one-way MANOVA. Assumptions of normality were met as assessed by Box’s M test of homogeneity of covariance (*p* = 0.815) and the Shapiro–Wilk test of multivariate normality (*p* = 0.628). MANOVA results indicated that the differences between participants did not demonstrate statistically significant effects on scores in the TGMD-3 (F(1, 3) = 0.764, *p* = 0.175).

While MANOVA results were found to be insignificant, one-way ANOVAs were conducted for each dependent variable. One-way ANOVA did not reveal significant differences based on group across the three different measurement conditions: locomotor raw skills, F(1, 20) = 1.81, *p* = 0.194, η^2^ = 0.083; ball skills raw skills, F(1, 20) = 0.566, *p* = 0.461, η^2^ = 0.028; GMQ, F(1, 20) = 0.729, *p* = 0.403, η^2^ = 0.035. Descriptive analyses were conducted to further explore group differences in locomotor skills, ball skills, and GMQ. Means and standard deviations for each dependent variable across groups are presented in Table 6. Those who only completed the baseline testing scored higher than those who remained in the program for both locomotor skills (M_diff_ = 3.63 points) and object control skills (M_diff_ = 2.18 points), while those who completed the program demonstrated higher overall GMQ (M_diff_ = 1.36 points). Small effect sizes were observed for locomotor skills (η^2^ = 0.083), ball skills (η^2^ = 0.028), and GMQ (η^2^ = 0.035), suggesting that the dropout rate for the current sample was not practically important.

## 4. Discussion

This research sought to address a gap in the FMS and physical literacy literature by examining the feasibility of an 8-week GBA community program for promoting physical literacy and FMSs in children aged 8–12. The primary aim of this study was to determine to what extent participation in the intervention influenced FMS development, as well as observe the effects of program duration and game-based design on skill acquisition. While statistical improvements were not observed between pre- and post-text assessments, descriptive trends suggest slight gains in both locomotor and ball skill scores.

Secondary analyses emerged due to dropout. These analyses aimed to assess between-group differences in FMS competence at baseline and were considered based on gender, age, and those who dropped out compared to those who remained in the program. Though insignificant, the results demonstrated that children at baseline demonstrated differences based on both gender and age, which was consistent with the literature.

Taken together, the findings highlight that the GBA intervention design may demonstrate potential to promote FMSs and physical literacy development, though further testing is required to confirm these trends. Preliminary trends indicate that a community-based, free, and low-budget GBA may offer a feasible approach to promote FMSs and physical literacy in underserved areas. Due to the pilot nature of this program and the small sample size, the findings should be interpreted cautiously.

Interventions that have improved FMS competence have varied in duration from approximately 4 weeks, to greater than 8 months [9,21,23]. Although the program spanned 8 weeks with slight improvements in FMSs, the once-weekly 90 min session may have lacked adequate frequency to promote meaningful motor skill development, consistent with prior findings suggesting that 2–3 sessions per week would yield the best FMS gains [9,21,23,25]. Though there were slight improvements, the lack of significant pre- and post-test effects suggests that the current structure and activities within the GBA may not have sufficiently promoted FMS improvements over the 8-week period. This underscores a need to further develop a GBA structure with regard to the type, frequency, and intensity of activities.

Higher levels of PA throughout life are underscored by higher levels of FMS competence developed during foundational years [7,8,9]. An average GMQ at baseline of 101.68 was found based on the average age of participants who completed baseline testing, which represents average levels of motor competence. Most participants demonstrated average or above-average FMSs [33], which were higher than global scores [3]. As the average age of participants was 9.82 years, children may have already achieved baseline proficiency in FMSs [4], thereby limiting the program’s effects and reducing the sensitivity of the TGMD-3 to detect meaningful gains in FMS competence. As typical FMS intervention research has taken place largely with preschool-aged children [9,21,23,25], younger children are more likely to exhibit more robust FMS improvement compared to older children, especially employing GBAs [19]. Future research should aim to examine and provide programming for younger cohorts, where greater effects on FMSs may be possible.

Ball skill scores demonstrated the most significant correlations, highlighting that initial proficiency in these skills may be related to improvements in other motor domains. Additionally, correlations and pairwise comparisons highlight that there are likely interdependencies between motor skill domains. In fact, there may be a hierarchy associated with FMS development, with locomotor skills developing first, followed by the object control domain [9]. The between-group comparison data, based on age, support this finding, with older children at baseline demonstrating higher object control skill scores compared to younger children.

While gender-based differences at baseline FMS testing were non-significant, descriptive trends (e.g., boys developing object control competence more quickly compared to girls) align with previous research findings [3,4,7,38]. Another study similarly found that although boys and girls consistently score differently in locomotor and ball skill domains, gender differences were not statistically significant [19]. The overall sample was skewed towards boys (*N* = 19), which may have influenced group dynamics and engagement with certain activities. For example, the higher frequency of boys in each group may have influenced the activities chosen during open play periods, which may have inadvertently favoured the existing strengths of those children.

These descriptive trends based on age and gender do highlight potential differences and may warrant exploration in a larger intervention. The gender imbalance in the current study likely restricted the ability to detect specific effects of gender and may have contributed to lower statistical power in analyses. Future research in GBAs, especially as this program continues to develop, should prioritize balanced recruitment efforts, and actively promote activity formats that ensure inclusive engagement regardless of gender.

To our knowledge, few studies have explored the impact of GBAs on FMS development among children [15,16,19]. Though the present research found that an 8-week GBA did not significantly impact FMS development, the results offer insight for future development of the program. A similar, school-based study focusing on FMS promotion through PA programming demonstrated analogous outcomes, suggesting that GBAs may have a small effect due to the small sample sizes [27].

### Limitations and Future Directions

Several limitations of the present study must be recognized. Most notably, there was a 42% dropout rate between baseline and post-test data collection. While this may be due to a number of factors (e.g., timing, parent/guardian conflict, child enjoyment, etc.), this did result in a low-powered study. In addition to these likelihoods, it is possible that dropout was not random; baseline TGMD-3 results did not reveal systematically different motor competence, but other factors such as perceived motivation, activity challenge, and/or fun may have influenced the desire of children to return to the program. Enrolment in the program was limited to 30 participants for a number of reasons: the number of instructors that were recruited, the resources that were available through the recreation facility at the institution, and the pilot nature of the program to explore initial engagement and interest from the community, as well as program delivery. The enrolment cap was also consistent with recommendations for pilot and feasibility programs [32]. To further explore the effects of GBAs through the PA program, higher initial enrolments would be beneficial to account for potential dropout over the course of the program. Additionally, future interventions and iterations of the program should focus on balanced recruitment to account for potential sampling bias in both gender- and age-based analyses and comparisons. For these reasons, there existed some context-specific factors that potentially limited the delivery and implementation of the program. Future development of the program should consider tracking dropout to better understand the rationale.

Another limitation of the present study was the lack of a control group. This limited the ability to observe whether the differences in TGMD-3 performance were due to the intervention itself, or if other factors (e.g., developmental progression, physical education or activity outside of the program) played a role. Similarly, there was no control or accounting for PA outside of the program. The program was designed as a free, accessible opportunity for children in a lower socioeconomic community to engage with PA and promote physical literacy. Further development of the program may allow for additional research to be conducted with these different components once the program is past the pilot stages [19,27,39]. As the program continues to develop, a waitlist control group may be beneficial, where children who are on the waitlist (e.g., beyond the enrolment cap) may be assessed to compare to the intervention group. This would allow for the program to maintain the accessible and community-based aims while also strengthening internal validity and providing a control-matched design.

A final limitation of the present research is the age of the participants. The program had children enrolled between the ages of 8 and 12 years. Children in this age group are likely to have already attained, at minimum, basic proficiency in FMSs [3,4], demonstrating a need for examination of lower age groups. Additionally, the TGMD-3 is designed for children between the ages of 3 and 10 years. The current participants’ ages exceeded this limit; however, it has been suggested that the TGMD-3 could be expanded for use with adolescents, with small adjustments to the skill execution [35]. Further exploration should focus on younger children, or other assessments that are designed for older age groups.

## 5. Conclusions

This study aimed to assess the feasibility of an 8-week GBA in a lower socioeconomic community to promote FMS development and physical literacy among 8- to 12-year-old children, with an emerged secondary aim of exploring whether children had significant differences prior to the intervention. While the final pre- and post-test comparisons were insufficiently powered to make meaningful observations regarding the effects of the program on FMS development, descriptive trends demonstrated increases in both locomotor and object control raw skill scores in the TGMD-3. Specifically, object control skills demonstrated the highest degree of change and between-group differences at baseline, with boys scoring slightly higher than girls and older children scoring higher than younger children. These trends align with the existing literature on FMS interventions and are useful for informing future program goals. These preliminary findings support initial implementation of a free, community-based GBA program to promote FMSs in underserved communities. Due to the limited statistical power in the present study and the lack of a control group, the findings must be interpreted cautiously. Further refinement of GBA programming, including data collection with a larger sample and control group, would be beneficial to explore the effectiveness of GBAs to promoting FMS development.

## Figures and Tables

**Table 1:** Demographic information for children enrolled in the program based on participation group.

	Younger Children	Older Children
**Gender**	Girls: 4 Boys: 11	Girls: 3 Boys: 8
**Age—M** **SD**	8.94 0.82	10.94 0.58
**Total**	15	11

*N* = 26; the number of participants who attended at least one session and completed at least one of the TGMD-3 testing sessions.

**Table 2:** Correlations among TGMD-3 subtests for all participants.

	1	2	3	4	5	6	7	8	9	10	11
1. Age Group	—										
2. PRE LS RS	0.004	—									
3. PRE LS SS	0.092	0.656 ***	—								
4. PRE BS RS	0.604 **	0.287	0.133	—							
5. PRE BS SS	0.111	0.164	0.115	0.512 *	—						
6. PRE GMQ	0.256	0.450	0.664 ***	0.304	0.598 ***	—					
7. POST LS RS	0.503	0.265	0.223	0.492	0.338	0.353	—				
8. POST LS SS	0.559	0.078	−0.282	0.570	0.241	−0.056	0.677 *	—			
9. POST BS RS	0.297	0.829 **	0.388	0.665 *	0.434	0.508	0.283	0.325	—		
10. POST BS SS	0.108	0.250	0.095	0.631 *	0.938 ***	0.586	0.375	0.391	0.350	—	
11. POST GMQ	0.354	0.212	−0.075	0.722 *	0.770 **	0.378	0.598	0.771 **	0.405	0.888 ***	—

Note: * *p* < 0.05, ** *p* < 0.01, *** *p* < 0.001. PRE: baseline scores; POST: post-test scores; LS: locomotor skills; BS: ball skills; GMQ: gross motor quotient; RS: raw scores; SS: scaled scores.

**Table 3:** Summary of TGMD-3 pre–post-test subtest change scores.

Category	Pre		Post		Mean Change	
	X	SD	X	SD	X	SD
Locomotor Raw Score	32.45	7.20	35.72	4.05	3.27	−3.15
Locomotor Scaled Score	10.45	1.04	10.27	0.46	−0.18	−0.57
Ball Skill Raw Score	37.45	7.75	40.36	6.10	2.91	−1.64
Ball Skill Scaled Score	10.36	0.81	10.27	0.64	−0.09	−0.162
Total Raw Score	69.91	14.95	76.09	10.16	6.18	−4.79
Gross Motor Quotient	102.36	4.20	101.63	2.80	0.73	−1.39

Note: *N* = 11; pre–post and change scores represent the average scores for those participants who completed both the pre- and post-test evaluations. Pre = pre-test; Post = post-test.

**Table 4:** Pairwise comparisons for TGMD-3 subtest scores.

Category		t-Statistic	*p*-Value	Effect Size	95% CI	
Measure 1	Measure 2				Lower	Upper
Pre LS Raw	Pre BS Raw	−2.106	0.061	−0.591	−1.274	0.029
Pre LS Scaled	Pre BS Scaled	0.447	1.000	0.333	−0.806	0.948
Post LS Raw	Post BS Raw	−2.651	0.024	−0.799	−1.469	−0.100
Post LS Scaled	Post BS Scaled	0.000	1.000	0.000	-0.791	0.791
Pre LS Raw	Post LS Raw	−1.494	0.166	−0.450	−1.063	0.182
Pre LS Scaled	Post LS Scaled	0.405	0.784	0.200	-0.645	0.825
Pre BS Raw	Post BS Raw	−1.631	0.113	−0.582	−0.877	0.034
Pre BS Scaled	Post BS Scaled	1.000	1.000	1.000	1.000	1.000
Pre GMQ	Post GMQ	0.405	0.784	0.200	−0.645	0.825

Note: For the parametric *t*-test, effect size is given by Cohen’s *d*. For the Wilcoxon test, effect size is given by the matched rank biserial correlation. Pre = pre-test; Post = post-test; LS = locomotor skill; BS = ball skill; GMQ = gross motor quotient.

**Table 5:** Pairwise comparisons for TGMD-3 raw test scores.

Skill	Baseline		Post-Test		t	*p*	Effect Size	95% CI	
	X	SD	X	SD				Lower	Upper
Run	9.000	2.720	6.000	1.612	3.146	0.010	0.949	0.214	1.652
Gallop	4.273	2.005	5.909	1.446	−2.206	0.949	−0.665	−1.309	0.005
Slide	4.455	1.968	6.727	1.104	−2.380	0.018	−0.944	−0.988	−0.762
Hop	6.182	1.250	6.545	2.067	−0.296	0.807	−0.111	−0.689	0.554
Horizontal Jump	5.182	2.040	5.545	1.293	−0.690	0.506	−0.208	−0.801	0.395
Skip	3.364	2.580	5.000	1.265	−2.104	0.062	−0.273	−1.273	0.030
Two-Hand Strike	6.818	1.601	7.455	1.368	−1.249	0.240	−0.377	−0.981	0.245
Forehand Strike	5.636	2.378	4.909	2.386	0.714	0.492	0.215	−0.388	0.808
Dribble	3.273	2.054	4.273	1.555	−1.158	0.274	−0.349	−0.951	0.269
Kick	4.909	1.446	5.364	1.629	−1.336	0.211	−0.403	−1.010	0.222
Two-Hand Catch	5.909	0.302	5.727	0.647	1.000	1.000	1.000	1.000	1.000
Overhand Throw	5.273	1.555	5.364	1.748	−3.985	0.003	−1.202	−1.972	−0.398
Underhand Throw	5.636	2.248	7.273	1.489	−0.142	0.890	−0.043	−0.633	0.549

Note: For the parametric *t*-test, effect size is given by Cohen’s *d*. For the Wilcoxon test, effect size is given by the matched rank biserial correlation.

**Table 6:** Descriptive statistics for between-group comparisons at baseline.

Variable	Group 1	Group 2	Effect Size (η^2^)	95% CI	
	Female	Male		Lower	Upper
Locomotor RS	34.83 (6.145)	34.06 (6.757)	0.003	0.000	0.172
Ball Skills RS	37.0 (7.211)	39.13 (6.692)	0.021	0.000	0.253
GMQ	103.33 (5.203)	101.06 (2.977)	0.077	0.000	0.350
	**Younger Children**	**Older Children**			
Locomotor RS	34.25 (6.151)	34.0 (7.15)	<0.001	0.000	0.000
Ball Skills RS	34.917 (6.07)	42.90 (4.70)	0.365	0.061	0.609
GMQ	100.83 (3.53)	102.7 (3.86)	0.065	0.000	0.333
	**Baseline & Post-Test**	**Only Baseline**			
Locomotor RS	32.46 (7.20)	36.09 (5.34)	0.083	0.000	0.357
Ball Skills RS	37.46 (7.75)	39.64 (5.69)	0.028	0.000	0.269
GMQ	102.36 (4.20)	101.00 (3.23)	0.035	0.000	0.284

Note: All scores are presented as mean and standard deviation unless stated otherwise. RS = raw skills; GMQ = gross motor quotient; CI = confidence interval.

## Data Availability

The raw data supporting the conclusions of this article will be made available by the authors on request due to ethical reasons.

## Supplementary Materials

The following supporting information can be downloaded at: https://www.mdpi.com/article/10.3390/children12070838/s1, Table S1: Activity Structure in the Physical Activity Program.

## Abbreviations

The following abbreviations are used in this manuscript:

MDPI	Multidisciplinary Digital Publishing Institute
DOAJ	Directory of open access journals
FMS	Fundamental movement skills
PA	Physical activity
GBA	Game-based approach
TGMD	Test of Gross Motor Development
