# Supplementary material for: Promoting Fundamental Movement Skills and Physical Literacy Among 8–12-Year-Old Children: Feasibility Insights from an 8-Week Pilot Program in Southwestern Ontario
Source: Children (Basel). 2025 Jun 25;12(7):838. doi: 10.3390/children12070838 (PMC12293679; doi:10.3390/children12070838)
Supplement: Supplementary file 1 [file children-12-00838-s001.zip › children-3669627-supplementary.pdf]

Table. Summary of intervention study activities.

Table S1. Activity Structure in the Physical Activity Program

Week	Focus	Younger Children Activities	Older Children Activities
1		TGMD Testing<br>Child-Led Games	TGMD Testing<br>Child-Led Games
2	Locomotor Skills	Octopus Tag<br>- Different rounds with different locomotor skills<br>Island Invasion<br>- Stealing treasure from middle using different locomotor skills per round<br>Hop the Hurdles Relay<br>- Using different locomotor skills – hopping, jumping – to complete a relay task<br>Locomotor Legends<br>- Different locomotor skills used based on colour cues from instructors	Tail Tag<br>- Pinnie/scarf in waist band<br>- Different rounds with different locomotor skills<br>Hop the Hurdles Relay<br>- Using different locomotor skills – hopping, jumping – to complete a relay task<br>Island Invasion<br>- Stealing treasure from middle using different locomotor skills per round<br>Stepping Stones Tag<br>- Obstacle course conducted if they are tagged in the game
3	Throwing Catching	Rubber Band<br>- One hand overhand throwing for distance<br>- Partner Catches the ball<br>Return to the Palace<br>- Throwing and catching to get team to move to the other side of the play space<br>Capture the Treasure<br>- Running team-based game to steal from the other team<br>Stepping Stones Tag<br>- Obstacle course conducted if they are tagged in the game<br>Legends of the Forest	Rubber Band<br>- One hand overhand throwing for distance<br>- Partner Catches the ball<br>Capture the Treasure<br>- Running team-based game to steal from the other team<br>Return to the Palace<br>- Throwing and catching to get team to move to the other side of the play space<br>Legends of the Forest<br>- Throwing game to knock over targets on other team's play space<br>Stepping Stones Tag

		- Throwing game to knock over targets on other team's play space	- Obstacle course conducted if they are tagged in the game
4	Dribble<br>Kicking	Jail Tag<br>- Running/locomotor activity<br>Four Corner Dribble<br>- Dribble a ball (hands) from one hoop to another to collect the most balls<br>All-Star Shootout<br>- Ball Shooting Game<br>Find and Dribble<br>- Use different locomotor movements to find a ball and dribble it to specific spot<br>Hungry Hungry Hippos<br>- Teams collect balls by dribbling in different ways from the middle to their net<br>Clear Your Yard<br>- Kick balls from one side of playing field to the other with targets	Colour Dribble<br>- Dribble around play area with specific colour cues<br>Hungry Hungry Hippos<br>- Teams collect balls by dribbling in different ways from the middle to their net<br>Clear Your Yard<br>- Kick balls from one side of playing field to the other with targets<br>Jail Tag<br>- Running/locomotor activity<br>All-Star Shootout<br>- Ball Shooting Game<br>Pass and Escape<br>- Tag based game incorporating ball passing and dribbling (hands)
5	Kicking	Dribble Octopus Tag<br>- Kicking soccer ball<br>Reaction Time Task<br>- One vs. One agility task while kicking soccer ball<br>Guard the Cone<br>- Pass, trap, and shoot soccer ball at a target<br>Minefield Tag<br>- Skipping/other locomotor task around the play area<br>Destroyers and Construction Workers<br>- Teams work against each other to run to pylon and flip them over	Minefield Tag<br>- Skipping/other locomotor task around the play area<br>Destroyers and Construction Workers<br>- Teams work against each other to run to pylon and flip them over or fix them depending on their role<br>Dribble Octopus Tag<br>- Kicking soccer ball<br>Reaction Time Task<br>- One vs. One agility task while kicking soccer ball<br>Capture the Treasure

		or fix them depending on their role<br>Capture the Treasure<br>- Running team-based game to steal from the other team	- Running team-based game to steal from the other team<br>Soccer Baseball<br>- Kicking and running activity, with some throwing
6	Throwing<br>Catching<br>Kicking	Tag Game<br>Red, Yellow, Green Light<br>- Ball control using the feet<br>Team Knock Downs<br>- Kicking ball towards target in obstacle activity<br>Boulder Ball<br>- Underhand Throws to knock down a target<br>- Team based activity<br>Swamp Ball<br>- Dodgeball Game involving overhand throws and catch rules	Ultimate Tag<br>Swamp Ball<br>- Dodgeball Game involving overhand throws and catch rules<br>Boulder Ball<br>- Underhand Throws to knock down a target<br>- Team based activity<br>Team Knock Downs<br>- Kicking ball towards target in obstacle activity<br>Gate Passing<br>- In pairs, kick ball to each other through gates, moving through play space
7	Throwing<br>Dribbling	Hurdle-Hop Relay<br>Clear Your Yard<br>- Ball Passing Game<br>Ball Dribbling Game<br>- Using different locomotor movements<br>Toss Tag<br>- Underhand Tossing<br>Child-Led Games	Hurdle-Hop Relay<br>All-Star Shootout<br>- Ball Shooting Game<br>Pass and Escape<br>- Tag based game incorporating ball passing and dribbling (hands)<br>Child-Led Games
8		TGMD Testing<br>Child-Led Games	TGMD Testing<br>Child-Led Games
